# Livin Regulates H2A.X^Y142^ Phosphorylation and Promotes Autophagy in Colon Cancer Cells via a Novel Kinase Activity

**DOI:** 10.3389/fonc.2019.01233

**Published:** 2019-11-14

**Authors:** Yang Ge, Bao-lin Liu, Jun-peng Cui, Shu-qiang Li

**Affiliations:** The Six Department of General Surgery, Shengjing Hospital of China Medical University, Shenyang, China

**Keywords:** Livin, autophagy, colon cancer cells, H2A.X, phosphorylation

## Abstract

**Objective:** To investigate Livin-mediated regulation of H2A.X^Y142^ phosphorylation via a novel kinase activity and its effect on autophagy in colon cancer cells.

**Methods:** The interaction between Livin and H2A.X was tested by immunoprecipitation. H2A.X–/– HCT116 cells were transfected with human influenza hemagglutinin (HA)-tagged WT or Y142F phospho-dead mutantH2A.X plasmids. GST-tagged recombinant Livin protein was used to perform *in vitro* pull-down experiment and kinase assay. H2A.X–/–Livin+/+ SW480 cells were co-transfected with H2A.X^WT^/H2A.X^Y142F^ plasmid and LC3 EGFP-tagged plasmid to explore whether H2A.X^Y142F^ was involved in Livin-mediated autophagy induced by starvation in colon cancer cells.

**Results:** Co-immunoprecipitation studies confirmed that Livin interacted with H2A.X and that it was phosphorylation dependent. *In vitro* kinase assay confirmed that Livin could phosphorylate H2A.X. Knockdown of Livin (Livin–/–) in SW480 cells or HCT116 cells canceled the starvation-induced autophagy in colon cancer cells; H2A.X–/–Livin+/+ SW480 cells transfected with H2A.X^WT^ activated autophagy induced by starvation while cells transfected with H2A.X^Y142F^ had no significant difference; Livin-H2A.X^Y142F^ axis activated autophagy in colon cancer cells through transcriptionally regulating *ATG5* and *ATG7*.

**Conclusion:** Livin promotes autophagy in colon cancer cells via regulating the phosphorylation of H2A.X^Y142^.

## Introduction

The nucleosome is a structure composed of a about 200 bp DNA molecule coiling outside and a histone octamer in the core, which is a basic structural unit of the chromosome. The histone octamer core consists of four histones identified as H2A, H2B, H3, and H49 (1). Multiple post-translational modifications, inclusive of phosphorylation, acetylation, methylation, and ubiquitination, of core histone proteins have been indicated to regulate histone modification and multitude of cellular functions (2–5). It has been shown that phosphorylation of H2A.X at tyrosine 142 (H2A.X Y142) is important in mediating response to DNA damage (6), whereas phosphorylation of H3 at tyrosine 41 (H3 Y41) is important in carcinogenic pathways (7)—highlighting the importance of tyrosine phosphorylation of core histone proteins as a key regulatory mechanism.

Inhibition of apoptosis is considered as one of the cardinal requirements of cancer cells to survive and propagate (8). Therefore, apoptosis and its regulatory mechanisms have become the focus of research in recent years. Livin is a crucial member of the inhibitor of apoptosis protein family (IAPs), it has a unique apoptosis inhibitory protein repeat (BIR) and a carboxy-terminal loop finger domain (RING), which binds to and suppress caspase protein, inhibiting apoptosis and promoting cell growth (9). Moreover, Livin can degrade secondary mitochondria derived activation of caspase (Smae), which is a novel mitochondrial protein, preventing its antagonism to IAPs and indirectly inhibiting caspases to delay normal cell apoptosis (10). Livin protein is mainly expressed in the cytoplasm in cancer cells. It had low or no expression in adult normal tissues, and high expression in embryonic tissues and some cancer tissues or cancer cell lines such as gastric cancer, breast cancer and colon cancer (11). Overexpression of Livin can modulate resistance to chemotherapy or radiotherapy in colon cancer (12).

Inhibition of Livin promoted the sensitivity of colon cancer cells to 5-fluorouracil (5-FU) via modulating the crosstalk between cellular apoptosis and autophagy (13). One of the central mechanisms of stress response is autophagy, which is involved in maintaining homeostatic balance by physiological clearance of organelles, proteins and is intricately involved in regulating cellular differentiation, defense against pathogens and nutritional starvation. Both stimulation and inhibition of autophagy has been shown to have therapeutic benefits in cancer cells, largely in a context-dependent fashion (14). The precise effect of Livin on autophagy in colon cancer cells and its underlying regulation mechanism are not known. In the present study, we investigated the effect of Livin on cellular autophagy in colon cancer cells and its potential role as a kinase regulating H2A.X^Y142^ phosphorylation.

## Materials and Methods

### Cell Culture

HT-29 cells, SW480 cells and HCT116 cells were purchased from ATCC (Shanghai, China). Cells were cultured in McCoy's 5A Medium (Gibco, USA). Transfection of expression plasmids or siRNAs were performed when cells were 40–60% confluent.

### Quantitative Polymerase Chain Reaction

Cells were harvested and 1 mL Trizol (Invitrogen, USA) was added to extract cellular RNA. Concentration of RNAs were measured by the optical density (OD) value using spectrophotometer (Thermo Scientific, USA). Reverse transcription of RNA (system: 10 μL) was performed to synthetize cDNA (Takara, Japan), which was used to template the real-time quantitative polymerase chain reaction (RT-qPCR) using the following settings: 95°C 2 min; (94°C 20 s, 60°C 20 s, 72°C 30 s) for 40 rounds.

### Western Blotting

One hundred microliters RIPA lysis buffer (Beyotime, Zhejiang, China) containing 1 μL PMSF (Sigma, USA) was used to extract the cellular proteins. Concentrations of proteins were measured by BCA kit (Thermo Scientific, USA). Antibodies against Livin (Cell Signaling Technology, USA), H2A.X (Abcam, USA), H2A.XY142F (Abcam, USA), GAPDH (Santa Cruz, USA), ATG5 (Cell Signaling Technology, USA), ATG13 (Cell Signaling Technology, USA), ATG14 (Cell Signaling Technology, USA), LC3-I/II (Abcam, USA) and β-actin (Santa Cruz, USA) were used at 1:1000 dilution.

### Dual Luciferase Reporter Assay

The dual-luciferase reporter gene assay kit (Promega, USA) was used according to the instruction manual. Colon cancer cells were cultured in 24-well plate and then co-transfected with pGL3-ATG5/pGL3-ATG7 plasmid and Livin-H2A.X^Y142F^/ Livin-H2A.X^Y142F^ for 8 h at 37°C.

### Co-immunoprecipitation Assay

Cells were harvested 48 h after transfection and lysed as above. Part of lysate was kept as input. One microgram of the corresponding antibodies were added to the rest of cell lysate, and incubate at 4°C overnight. 15 μL 2 × SDS loading buffer was added to immunoprecipitated beads, boiled for 5 min, and then resolved by SDS-PAGE and processed for western blotting analysis.

### Long-Lived Protein Degradation Assay

The whole culture medium containing 1.0 μCi/mL L-[3,5-^3^H]-tyrosine (Institute of Atomic Energy, Beijing, China) was cultured for 48 h, then the cells are washed with 2 mL of ice-cold Hank's balanced salt solution (containing 2 mmol/L non-isotopically labeled tyrosine) twice, followed by culturing the cells for 2 h in DMEM (containing 10% FBS and 2 mmol/L non-isotopically labeled tyrosine) to degrade short-lived proteins. Cells were washed twice with 2 mL of ice-cold Hank's balanced salt solution (containing 2 mmol/L non-isotopically labeled tyrosine) and measure the culture medium and intracellular L-[3,5-^3^H]- tyrosine using a liquid scintillation counter. Long-life protein degradation rate = L-[3,5-^3^H]-tyrosine content in culture medium/(L-[3,5-^3^H]-tyrosine content in culture solution+ intracellular).

### Recombinant Proteins and *in vitro* GST Pull-Down Experiment

Livin was inserted in the pGEX-5X-1 vector (Amersham Biosciences Corp) to generate GST-Livin construct. The H2A.X wild-type DNA sequence was inserted into the pET-46Ek/LIC vector (Novagen, Madison, WI, USA) following manufacturer guidelines to generate His-H2A.X wild-type construct. The Y142F mutant was generated by site-directed mutagenesis. Both GST-Livin and His-tagged wild-type or Y142F H2A.X were expressed in E. coli and purified according to manufacturer protocol (Thermo Scientific, USA). For pull-down assays, GST-Livin was bound to glutathione beads followed by wash to get rid of non-specific binding. Indicated concentrations of wild-type His-H2A.X was then added to the GST-Livin bound glutathione beads and incubated at room temperature for 2 h followed by 6 washes, before being eluted using SDS-loading buffer.

### *In vitro* Kinase Assay

Recombinant wild-type or Y142F mutant His-tagged H2A.X proteins (400 ng) were incubated with 100 ng of GST-Livin at 30°C for 30 min in 1× kinase buffer containing 10 μmol/L of unlabeled ATP or 10 μCi[γ-^32^P]ATP. Samples were boiled and then resolved by SDS-PAGE and visualized by autoradiography using Phosphor-Image Screens (Tokyo, Japan).

Post-autoradiography blots were coomassie stained to confirm equivalent amounts of protein loading across lanes.

### Statistical Analysis

Statistical analysis was performed by GraphPad Prism 6.0 (New York, USA). Data was represented as mean ± SD. Two-way ANOVA was used to test differences between groups. *P* < 0.05 was considered as statistically significant.

## Results

### Livin Promoted H2A.X^Y142ph^ Expression in Colon Cancer Cells

**Figure 1:**
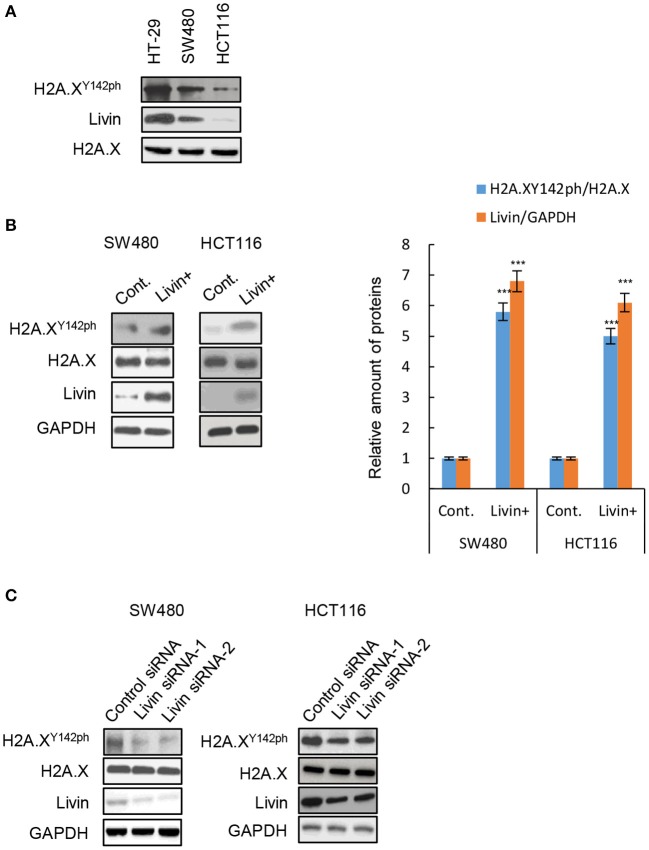
Livin promoted H2A.X^Y142ph^ expression in colon cancer cells. **(A)** The protein levels of H2A.X^Y142ph^ and Livin in three different cell lines of colon cancer were analyzed by western blotting; **(B)** The SW480 cells and HCT 116 cells were transfected with Livin plasmid to over-express Livin protein (Livin+ group), cells transfected with empty plasmid were used as control (Cont. group), then the protein levels of H2A.X^Y142ph^ and Livin were tested by western blotting, and the gray value statistics were measured by Image J software. Data were shown as (Mean ± SD). ^***^P < 0.001; **(C)** Two Livin siRNAs targeting different sites were transfected into SW480 cells and HCT 116 cells, the protein levels of H2A.X^Y142ph^ and Livin in three different cell lines of colon cancer were analyzed by western blotting.

We measured the protein expression of Livin and phosphorylated H2A.X^Y142^ (H2A.X^Y142ph^) in three different types of colon cancer cells. We found that the levels of Livin in HT-29 cells and SW480 cells were obviously higher compared to HCT116 cells (Figure 1A). Interestingly, the expression of H2A.X^Y142ph^ mimicked the pattern observed for Livin (Figure 1A). We next overexpressed Livin in SW480 cells and HCT116 cells. Compared to the control group, H2A.X^Y142ph^ expression increased following overexpression of Livin in both HT-29 and HCT116 cells (Figure 1B). Conversely, knockdown Livin using siRNA resulted in reduction of H2A.X^Y142ph^ levels (Figure 1C). Taken together, these data indicated that Livin promoted the expression of H2A.X^Y142ph^ in colon cancer cells.

### Livin Physically Interacted With H2A.X in Colon Cancer Cells

**Figure 2:**
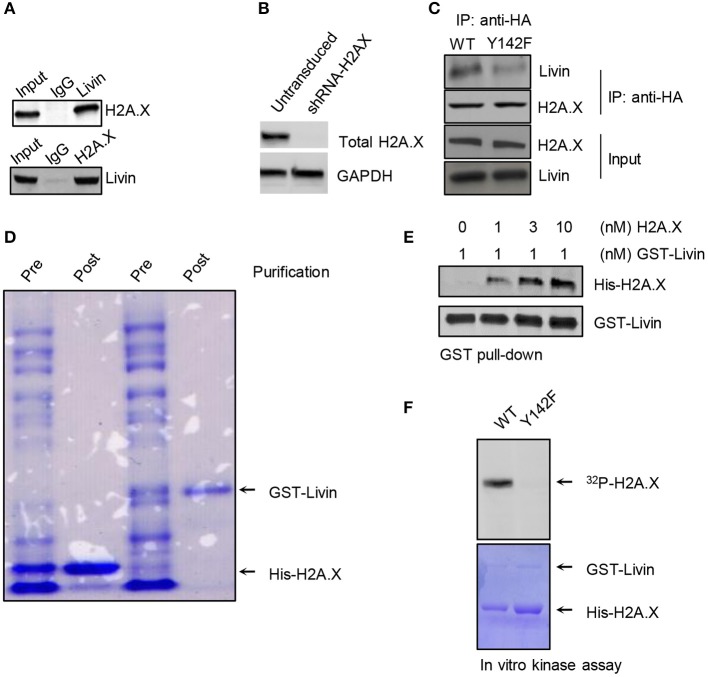
Livin directly interacts with H2A.X and phosphorylates it at Y142. **(A)** The interaction of Livin with H2A.X was demonstrated by Immunoprecipitation in SW480 cells, the binding of H2A.X and Livin was detected by western blotting; **(B)** HCT116 cells transduced with shRNA targeting 3′-UTR of H2A.X and selected using puromycin. Selected cells were immunoblotted to confirm knockdown of H2A.X. **(C)** HCT116 cells harboring shRNA-H2A.X were transfected with H2A.X^WT^ or H2A.X^Y142F^ plasmid tagged with HA. The immunoprecipitation was performed and the bonding band of H2A.X^WT^/ H2A.X^Y142F^ and Livin was tested by western blotting; **(D)** Coomassie stain to confirm effective expression and purification of GST-Livin and His-H2A.X; **(E)** *in vitro* pull-down assay was done to test the association between the Livin plasmid tagged with GST and His-H2A.X; **(F)** *in vitro* kinase assay using recombinant Livin and wild-type or Y142F mutant H2A.X. HA, human influenza hemagglutinin; GST, Glutathione S-transferase; WT, wild type; H2A.X^Y142ph^, H2A.X^Y142^ phosphorylation.

To further explore the regulation mechanisms of Livin on H2A.X^Y142ph^, we probed interaction of Livin with H2A.X by reversible immunoprecipitation assay in SW480 cells (Figure 2A). Immunoprecipitation using either Livin or H2A.X antibody could pull-down H2A.X and Livin, respectively (Figure 2A), We next determined if interaction of Livin or H2A.X wad dependent on H2A.X^Y142ph^. Given that HCT116 cells had the least expression of H2A.X among the colon cancer cell lines tested (Figure 1A), we used HCT116 cells for this experiment. Endogenous H2A.X was stably knocked down in HCT116 cells using shRNA-targeting the 3′-UTR of H2A.X (Figure 2B). These cells were then transfected with human influenza hemagglutinin (HA)-tagged WT or Y142F phospho-dead mutant H2A.X plasmids. As shown in Figure 2C, the amount of Livin bound to H2A.X^Y142F^ was markedly less than that bounding to H2A.X^WT^. This finding indicated that the interaction of Livin with H2A.X in these cells was dependent on H2A.X^Y142^ phosphorylation. Even though these assays suggested that Livin interacted with H2A.X, the interaction can be mediated by other factors interacting with either H2A.X or Livin. Hence, to determine if Livin and H2A.X interacted directly, we generated recombinant GST-Livin and bought H2A.X (ab134863, Abcam, Waltham, MA) (Figure 2D). *In vitro* binding assays confirmed that Livin interacted directly with H2A.X (Figure 2E).

### Livin Functions as a Kinase in Phosphorylating H2A.X^Y142^

Based on our results, we hypothesized that Livin had kinase activity which could phosphorylate H2A.X^Y142^. *In vitro* kinase assay showed that recombinant Livin could phosphorylate wild type H2AX but not Y142F phospho-dead mutant H2AX (Figure 2F). This result indicated that the site of Y142 in H2A.X was the specific target of Livin. These results indicated that Livin is the kinase that is phosphorylating H2A.X at Y142.

### Livin Was Necessary for Starvation-Induced Autophagy in Colon Cancer Cells

**Figure 3:**
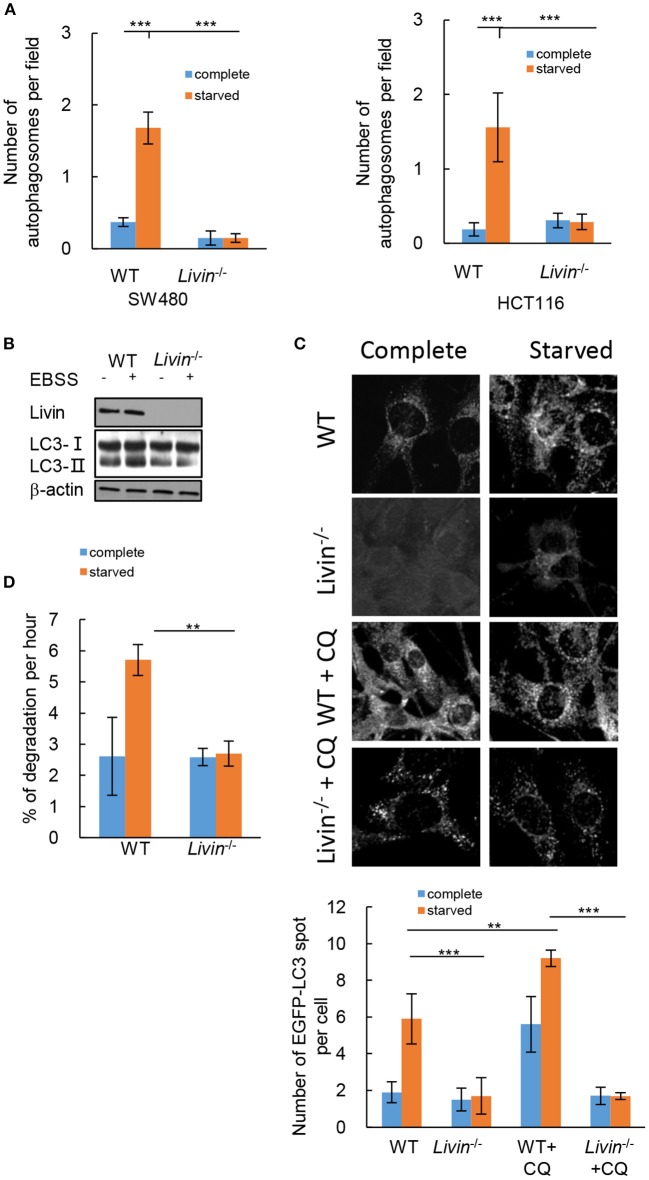
Livin was necessary for starvation-induced autophagy in colon cancer cells. **(A)** SW480 cells and HCT116 cells were treated with complete medium or EBSS for starvation. The number of cytoplasmic autophagosomes was measured by TEM, 20000X, scale bar = 2 μm. Each field was randomly selected for the statistics of autophagosomes, and the average of 6 fields of view was calculated. Data were shown as (Mean ± SD). ^**^P < 0.01, ^***^P < 0.001; **(B)** WT and Livin^−/−^ HCT116 cells were cultured in complete medium or EBSS. The protein expressions of LC3-I/LC3-II, Livin, and β-actin were evaluated by western blotting; **(C)** Livin^−/−^ and WT HCT116 cells were transfected with EGFP plasmid which tagged LC3, cells were cultured in complete medium or EBSS for 2 h, then 20 μM CQ was added for 6 h. The fluorescence of EGFP-LC3 puncta was measured, 80X, scale bar = 50 μm. Each field was randomly selected for the statistics of fluorescence intensity, and the average of 6 fields of view was calculated. Data were shown as (Mean±SD). ^**^P < 0.01, ^***^P < 0.001; **(D)** The long-lived protein degradation experiment was performed to measure the degradation rate of long-lived proteins in *Livin*^−/−^ HCT116 cells and WT HCT116 cells. Data were shown as (Mean ± SD). ^**^P < 0.01, ^***^P < 0.001; EBSS, Earle's balanced salt solution; TEM, transmission electron microscope; LC3, protein 1 light chain 3; EGFP, enhanced green fluorescent protein; CQ, chloroquine.

To evaluate the effect of Livin on autophagy in colon cancer cells, transmission electron microscopy (TEM) was performed to observe the number of cytoplasmic autophagosomes. The autophagy was induced by starvation when culturing in Earle's balanced salt solution (EBSS). Comparing to the cells culturing in complete medium, the starved group showed a significant increase of autophagosomes in both SW480 cells and HCT116 cells (Figure 3A). The protein level of LC3-II, which is a classical marker for mature autophagosome, was also up-regulated by starvation in WT HCT116 cells (Figure 3B). The protein level of Livin was increased by starved stimulation in WT HCT116 cells (Figure 3B). The fluorescence assay presented that the number of cytoplasmic EGFP-tagged LC3 puncta markedly increased by starvation (Figures 3C,D). However, siRNA-mediated knockdown of Livin (*Livin*^−/−^) in SW480 cells or HCT116 cells canceled all these changes induced by starvation (Figures 3A–C). These results indicated that Livin was necessary for starvation-induced autophagy in colon cancer cells.

Autophagy is a dynamic process, the accumulation of cytoplasmic LC3-II cannot represent the activation of autophagy, which can also be caused by blocking the degradation of autophagosomes (15). To decide whether the increased autophagosomes and LC3-II protein induced by starvation in WT HCT116 cells was a consequence of activated autophagy pathway, we evaluated the autophagic flux. After the number of EGFP-tagged LC3 puncta markedly increase by starvation in WT HCT116 cells, additional intervention of chloroquine (CQ) was performed, which was an inhibitor of late autophagy by suppressing lysosomal function and autophagosome degradation (16). The data showed the number of EGFP-tagged LC3 puncta further increased with CQ intervention in WT HCT116 cells, while it still had no change in *Livin*^−/−^ HCT116 cells (Figures 3C,D). Moreover, the long-lived protein degradation experiment demonstrated that the degradation rate of long-lived proteins in *Livin*^−/−^ HCT116 cells was inhibited comparing to WT HCT116 cells, indicating that Livin deficiency could suppress the autophagic flux (Figure 3D).

### H2A.X^Y142ph^ Was Necessary for Livin-Mediated Autophagy in Starvation-Stimulated Colon Cancer Cells

**Figure 4:**
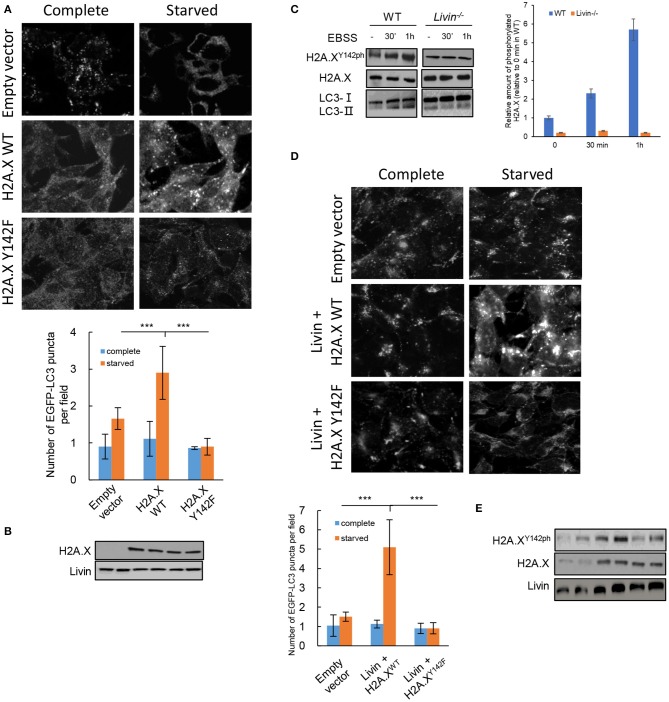
H2A.X^Y142ph^ was necessary for Livin-mediated autophagy in starvation-stimulated colon cancer cells. **(A,B)** H2A.X^−/−^Livin^+/+^ SW480 cells were co-transfected with H2A.X^WT^/H2A.XY^142F^ plasmid and LC3 EGFP-tagged plasmid. After starvation, the fluorescence of EGFP-LC3 puncta was measured, 80X, scale bar = 50 μm. Each field was randomly selected for the statistics of fluorescence intensity, and the average of 6 fields of view was calculated. Data were shown as (Mean ± SD). ^***^P < 0.001. The protein levels of total H2A.X and Livin was measured by western blotting **(B)**; **(C)** The kinase activity of Livin for H2A.X^Y142^ in SW480 cells with starved stimulation for 30 min and 1 h was evaluated by protein levels of H2A.X^Y142ph^; **(D,E)** WT SW480 cells were transfected with Livin plasmid to overexpress Livin (Livin+) after starvation. The fluorescence of EGFP-LC3 puncta was measured, 80X, scale bar = 50 μm. Each field was randomly selected for the statistics of fluorescence intensity, and the average of 6 fields of view was calculated. Data were shown as (Mean ± SD). ^***^P < 0.001. The protein levels of H2A.X^Y142ph^, H2A.X and Livin were measured by western blotting **(E)**.

To explore whether H2A.X^Y142ph^ was enrolled in the Livin-mediated autophagy induced by starvation in colon cancer cells, *H2A.X*^−/−^*Livin*^+/+^ SW480 cells were co-transfected with H2A.X^WT^ or H2A.X^Y142F^ plasmid and LC3 EGFP-tagged plasmid. After starvation, the number of EGFP-LC3 puncta in H2A.X^Y142F^ group had no significant difference with empty vector group, and the number of EGFP-LC3 puncta in H2A.X^WT^ group was obviously higher than both of them (Figures 4A,B). This result suggested that H2A.X^Y142ph^ had an important effect on autophagy induced by starvation in colon cancer cells.

Then we tested the kinase activity of Livin for H2A.X^Y142^ in SW480 cells with starved stimulation. H2A.X^Y142ph^ expression in WT group was obviously increased after starvation for 30 min and 1 h, while no significant variations of H2A.X^Y142ph^ were found in *Livin*^−/−^ group after starvation for 30 min and 1 h (Figure 4C). This data illustrated that Livin was an indispensable phosphorylated kinase for H2A.X^Y142^ under autophagy induced by starvation in colon cancer cells. To further confirm the role of H2A.X^Y142ph^ in Livin-mediated autophagy, WT SW480 cells were transfected with Livin plasmid to overexpress Livin (Livin+) after starvation. We found a markedly increase in H2A.X^Y142ph^ protein keeping pace with the activated autophagy level, as assessed by increased number of EGFP-LC3 puncta (Figures 4D,E). However, H2A.X^Y142ph^ expression downregulated through expressing H2A.X^Y142F^ and the autophagy activity decreased (Figures 4D,E). Taking together, these data demonstrated that overexpression of Livin could activate autophagy in starvation-stimulated colon cancer cells via H2A.X^Y142^ phosphorylation.

### The Livin-H2A.X^Y142ph^ Axis Activated Autophagy in Colon Cancer Cells Through Transcriptionally Regulating ATGs

**Figure 5:**
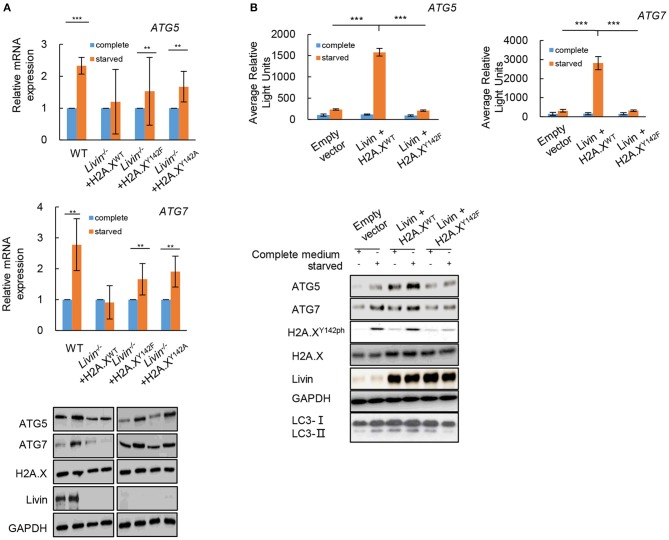
The Livin-H2A.X^Y142ph^ axis activated autophagy in colon cancer cells through transcriptionally regulating ATGs. **(A)** Livin^−/−^ SW480 cells were transfected with H2A.X^Y142A^ or H2A.X^Y142F^ mutant which were similar with the phosphorylated formation of H2A.X^Y142^. After starvation, the mRNA levels of ATG5 and ATG7 were measured by RT-qPCR, and the protein levels of ATG5, ATG7, H2A.X and Livin were tested by western blotting. Data were shown as (Mean ± SD). ^**^P < 0.01, ^***^P < 0.001; **(B)** The pGL3-ATG5 and pGL3-ATG7 plasmid were constructed for the luciferase assay. The WT SW480 cells were co-transfected with pGL3-ATG5/ pGL3-ATG7 plasmid and H2A.X^WT^/H2A.XY^142F^ plasmid, then the average relative lights units were used to evaluate gene transcriptional activity. Data were shown as (Mean ± SD). ^**^P < 0.01, ^***^P < 0.001. The protein levels of ATG5, ATG7, H2A.X, Livin and LC3I/LC3II were tested by western blotting.

Further investigation was done to test whether the activated effect of Livin-H2A.X^Y142ph^ axis on autophagy in colon cancer cells was through transcriptionally regulating ATGs. Firstly, the mRNA levels of ATG5 and ATG7 were markedly up-regulated in WT SW480 cells after starvation (Figure 5A), which did not change in *Livin*^−/−^ SW480 cells after starvation. When the *Livin*^−/−^ SW480 cells were transfected with transfected with the phospho-dead H2A.X^Y142F^ or H2A.X^Y142A^ mutants, the mRNA levels of ATG5 and ATG7 were markedly up-regulated after starvation (Figure 5A). The protein levels of ATG5 and ATG7 showed the same changes in each group as the mRNA levels (Figure 5A). This result indicated that the Livin-H2A.X^Y142ph^ axis could significantly increase both mRNA and protein expressions of ATGs under starved stimulation.

Then the pGL3-ATG5 and pGL3-ATG7 plasmid were constructed for the luciferase assay. The result showed that the Livin-H2A.X^Y142ph^ axis could directly target ATG5 and ATG7 in colon cancer cells under starvation, while Livin-H2A.X^Y142F^ axis had no combination with ATG5/ATG7 in colon cancer cells under starvation (Figure 5B). Moreover, the conversion of LC3-I to LC3-II protein promoted by transcriptional activation of ATGs was induced by the Livin-H2A.X^Y142ph^ axis, while Livin-H2A.X^Y142F^ axis canceled the changes (Figure 5B). These data suggested that Livin-mediated autophagy was through H2A.X^Y142ph^-induced transcriptional activation of ATGs.

## Discussion

The anti-apoptotic mechanism of the AlPs family was mainly caused by inhibition of caspase protease and its cascade activation reaction. Livin also acted mainly through this pathway, and it also had other anti-apoptotic pathways (17). A study found that intact Livin relied on the BIR domain to bind to downstream molecules that mediating apoptosis, including the activated forms of caspase-3 and caspase-7, and inhibited their activity (18). Another study found that Livin bind to TAK1 binding protein 1 (TAB1) and activates TGF-β activated kinase 1 (TAK1), which was a co-reactant of TAB1. TAB1 itself had no activation effect on TAK1, but promoted TAK1-mediated c-Jun N-terminal kinases 1 (JNK1) activation, which was important for Livin to counteract tumor necrosis factor-α (TNF-α) and Interleukin-1beta-converting enzyme (ICE)-mediated apoptosis and was an apoptotic mechanism independent of the caspase pathway (19). *In vitro* experiments confirmed that after knocking out the Livin gene of lung cancer SPC-A1 cell line, caspase-3 protein level was down-regulated, but there was no significant change in mRNA level (20). At the same time, phosphorylated JNK1 protein expression was reduced, while the mRNA and total protein levels of JNK1 were almost unchanged. This study further confirmed that Livin could activate the JNK1 signaling pathway to exert anti-apoptotic effects.

Many studies from Livin discovered to date have shown that this gene is expressed in a variety of malignancies. Livin expression was found in both renal cell carcinoma and normal kidney tissues, but its expression in cancer tissues was significantly higher, which was statistically significant (21). In addition, in a study focusing on the relationship between Livin and gastric cancer, Livin gene was overexpressed in gastric cancer cells and was closely related to tumor differentiation and lymph node metastasis (22). Apoptosis could be induced by shRNA inhibition of Livin expression, which might be a new targeted therapy for gastric cancer. In cervical cancer, the two subtypes of Livin were highly expressed and closely related to clinical stage, and positively correlated with the expression of Bcl-2, indicating that they played a coordinating role in the carcinogenesis of cervical cancer (23). In our present study, we explored the effect of Livin on autophagy in colon cancer cells. As we known, autophagy was a different type of programmed cell death differing from apoptosis. The data showed that Livin was necessary for starvation-induced autophagy in colon cancer cells. Knockdown of Livin (Livin–/–) in SW480 cells or HCT116 cells canceled all these changes induced by starvation (Figures 3A–C). Further investigation should be performed to prove the effect of Livin-mediated autophagy on cellular apoptosis in colon cancer cells.

Among the four core histones, H2A had the most variants, of which H2A.X accounted for <10%, but there have been many reports of H2A.X (24). Fragmentation of the DNA double strand could result in phosphorylation of the C-terminal serine site of H2A.X, which played an important role in specifically recognizing the C-terminus of H2A.X by mediator of DNA damage checkpoint protein 1 (MDC1), allowing more DNA repair factors aggregation (25). By analyzing the structure of the after the C-terminal domain of a breast cancer susceptibility protein (BRCT) in H2A.X, scientists have found that tyrosine 142 residue (Y142) was its terminal residue and had crucial effect on the binding of H2A.X to MDC1 (26). Under normal physiological conditions, Y142 was phosphorylated and dephosphorylation occurred during DNA damage. The phosphorylation of Y142 site in H2A.X could determine the cellular fate under DNA damage (27). On the one hand, when the DNA damage was moderate and the repair of DNA damage seemed workable, the dephosphorylation of Y142 occurred, accompanying by the phosphorylation of S139 (28). This variation led to the accumulation of DNA repair factors including MDC1. On the other hand, when the DNA damage was serious and had no repairability, the phosphorylation of Y142 still remained and the phosphorylation of S139 reduced, which resulting in the activation of JNK1, and the cellular fate went to apoptosis (29). A recent study reported that under the radiated exposure, there was a significant increase of JNK1-H2A.X interaction (30). In the present study, we found a markedly increase in H2A.XY^142ph^ protein keeping pace with the activated autophagy level. However, H2A.X^Y142ph^ expression down-regulated through expressing H2A.X^Y142F^ and the autophagy activity decreased (Figure 4C). The Livin-H2A.X^Y142ph^ axis activated autophagy in colon cancer cells through transcriptionally regulating ATGs. These results indicated that Livin had kinase activity for H2A.X^Y142^ phosphorylation. Whether Livin could influence DNA repair in colon cancer cells through H2A.X^Y142^ phosphorylation should be further investigated. It has been shown that H2A.X can be phosphorylated at other tyrosine residues too. Whether Livin is the kinase that phosphorylates at the other tyrosine residues and their function in autophagy need to be investigated in future studies.

In general, this study clarified the regulation of Livin in H2A.X^Y142^ phosphorylation which promoted autophagy in colon cancer cells. This finding provided a new sight into the pathogenesis of colorectal cancer, and may be a new target for medical therapy.

## Data Availability Statement

All datasets for this study are included in the article/supplementary material.

## Author Contributions

YG and SL designed experiments. BL carried out experiments. JC analyzed experimental results. YG wrote the manuscript. SL approved the manuscript.

### Conflict of Interest

The authors declare that the research was conducted in the absence of any commercial or financial relationships that could be construed as a potential conflict of interest.
